# Is it ‘just’ trauma? Use of trauma-informed approaches and multi-agency consultation in mental healthcare of looked after children

**DOI:** 10.1192/bjb.2023.3

**Published:** 2023-12

**Authors:** Nic Miller, Sreekumar Nair, Pallab Majumder

**Affiliations:** 1 University of Nottingham, Nottingham, UK; 2 Nottinghamshire Healthcare NHS Foundation Trust, Nottingham, UK

**Keywords:** Trauma, childhood experience, education and training, developmental disorders, qualitative research

## Abstract

This article presents three case studies of patients that a child and adolescent mental health service (CAMHS) have supported and its purpose is to encourage discussion of two key learning points. The first of these is the utility of developmental trauma as an approach for children with mental health presentations. The second centres on the importance of multi-agency working when working with young people, principally those within the UK's local authority care system (‘looked after children’), who have had traumatic experiences in order to enhance positive outcomes. We also want to encourage consideration of the implications of developmental trauma for current core CAMHS therapeutic models in an attempt to reach beyond the often held narrative that the trauma formulation implies there is ‘just trauma, no mental illness’.

As a society and a healthcare system we are becoming increasingly ‘trauma-informed’. This is in part due to more research indicating a strong correlation and causal link between adverse childhood experiences and mental ill health, homelessness, antisocial behaviour and chances of being in prison. Furthermore, when accounting for confounding factors, many studies in this field have linked specific categories of trauma in early years to specific categories of psychopathology in adulthood.^1–3^

The growing adult mental health crisis (and also many physical health issues) is therefore being increasingly linked with a need for ensuring improved outcomes in child and adolescent mental health services (CAMHS), particularly for those whose pathology is linked to early life trauma or adverse childhood experiences.^4^

A government report from 2018, however, highlighted that at present there is a ‘fragmented and highly variable approach to early intervention across England’.^5^ The question therefore remains ‘What does a good early intervention look like when a young person has experienced trauma during their early years?’ (under 10 years old for the purpose of this paper). The government report could not identify an adequate evidence base for early intervention work or identify a cohesive national strategy in early-years groups.^5^

Traumatic experiences are particularly pervasive among children in foster care (‘looked after children’). In 2018–2019, 63% of looked after children in England were in care because of abuse or neglect.^6^ In 2019, the Strengths and Difficulties Questionnaire (SDQ) was administered for 78% of children in the UK who had been in local authority care for over 12 months: 39% had reported scores indicating cause for concern and a further 13% had scores indicating borderline cause for concern.^7^ Working with looked after children to improve mental health outcomes in the early years is therefore an important area to consider when tackling the issue of adverse childhood experiences and adult psychopathology.

The implications of developmental trauma are often recognised, but this understanding is rarely used in therapeutic interventions within the traditional treatment pathways of core CAMHS. This leads to often unhelpful narratives such as it is ‘just trauma, not mental illness’ in children referred to CAMHS. Given the high numbers of looked after children who have experienced adverse childhood experiences, it is therefore also important to consider how the usual linear CAMHS pathways and diagnostic boxing fails to address the complex and everchanging care systems in which these children and young people find themselves.

The National Institute for Health and Care Excellence (NICE) highlights in its guidance on working with looked after children that there is a ‘need for those in the care network to form a relationship with the child or young person and with other practitioners within the network and use shared decision making'.^8^

Therefore, in this paper, we are presenting three case studies with the aim of demonstrating real-world examples of when using a trauma-informed approach has been successful and when care has been less successful when not engaging with this approach.

In line with the aforementioned NICE guidance, we want to highlight examples where a multi-agency approach has succeeded in helping the recovery of young people and demonstrate the utility of an indirect therapeutic approach when working with children with developmental trauma-related presentations. We also give examples of clinical situations where a trauma-informed lens has proven to be beneficial in the therapeutic approach, moving beyond the usual linear models in CAMHS. Within this we aim to show how looked after children are often let down by simplistic diagnostic boxes that do not account for their complex needs.

Written informed consent was obtained from the parent or legal guardian for all young people in the following case studies. All names have been changed.

## Case studies

### Sarah

Sarah is a 15-year-old who has been with the CAMHS looked after children (LAC) team since 2020. Sarah was adopted at the age of 2 and had been with the same adoptive parents since this age. However, in 2010 the adoptive parents separated and Sarah lived with her adoptive mother. Sarah's adoptive mother had another partner and child in 2016 but broke up with that partner in 2017.

The birth family environment had been unsettled throughout Sarah's very early years, with her birth mother's partners frequently changing. Sarah's biological older brother, who had been adopted by the same family as Sarah, had been displaying difficulties from a young age. It was reported that he had difficulties communicating his emotions and would often be unkind and hurtful towards Sarah, which she in turn struggled to understand and manage. Sarah said that she believed she had been put up for adoption as her parents were too young and probably abusive, as her brother had ended up in hospital when younger.

The difficulties with her brother were a persistent cause of distress throughout Sarah's history, until he moved out to live with a partner in recent years. However, on his return to the family home distress increased again and in discussions following her overdose attempts, Sarah frequently mentioned her brother as a potential trigger.

In March 2018 Sarah presented to the local CAMHS team with a risk of self-injury, thoughts of cutting with intent to end life and an incident in which she had a rope with which she intended to hang herself. Sarah's history up to this point included 11 attempted overdoses with paracetamol or other medication and 4 incidents of ingesting metallic (usually sharp) or chemically risky foreign bodies (a drawing pin, pencil sharpener blades, a sewing pin, watch batteries). On every incident of attempted suicide or self-harm Sarah would notify her adoptive mother either via a text (which tended to be an apology) or social media post or leaving out objects indicating the attempt, such as a bowl with vomit in on one occasion.

Sarah also frequently mentioned concerns about rejection by friends because of her sexuality, and some of the overdoses had been precipitated by fall outs with friends or break-ups.

Sarah attended both dialectical behavioural therapy (DBT) (an 8-week programme in 2018–2019) and cognitive–behavioural therapy (CBT) (7 sessions in late 2019) for a provisional diagnosis of anxiety. However, these appear to have made no change to her self-harming behaviour or suicidal ideation. In July 2020 a course of dyadic developmental psychotherapy (DDP) was commenced which is ongoing at the time of writing. The therapy helps the child or young person to experience relational safety and develop the ability to trust adults.

Sarah and her adoptive mother began to engage well in DDP sessions. However, there was an escalation in self-harm in the months after starting this therapy which led to frequent admissions to the paediatric ward in the local hospital. During assessments, Sarah described feeling consistently low in mood and therefore a course of an antidepressant (fluoxetine) was trialled during this period. Sarah did not find the medication beneficial; in fact, she felt that her urge to self-harm increased while on medication and therefore it was discontinued.

Keeping Sarah safe in the family setting became more and more challenging and she became very opportunistic in terms of accessing means of self-harm. In-patient admission was discussed but this was not deemed appropriate as although it would keep her safe in a psychiatric setting in the short term, it was unlikely to serve any long-term benefits.

At the time of writing Sarah is in a residential placement where she can continue to access DDP while her immediate safety has also been ensured. In this setting, Sarah has demonstrated longer periods of stability, better engagement in education and social activities and subsequent reduction in self-harm behaviour.

### Helena

Helena is an 11-year-old girl whose biological mother reportedly had intellectual disability and autism. The local health and social care teams were involved with her biological mother prenatally.

The biological mother was described as very vulnerable and reportedly lived a very chaotic lifestyle. It is considered possible that Helena witnessed inappropriate sexual activity and suffered severe neglect while young. At 16 months old she was taken into temporary foster care until the age of 3, when she was adopted.

Helena presented to CAMHS via the community paediatrician at the age of 11 with an increase in emotional dysregulation and challenging behaviour at home. It was reported that she would display significant verbal and physical aggression towards her adoptive mother. Her sleep was poor despite a clear bedroom routine. She would wake very early and be unable to get back to sleep.

Helena's developmental milestones were all delayed and some degree of intellectual disability was also suspected. She received support from the local community paediatric team from the age of 3. She was given diagnoses of autism spectrum disorder (ASD), attention-deficit hyperactivity disorder (ADHD) and sensory processing disorder in the initial few years of their involvement. At the age of 7, a private clinical psychologist further diagnosed pathological demand avoidance. There was possibility of prenatal exposure to alcohol, but fetal alcohol syndrome was ruled out. Helena had tried some sensory integration work and dance therapy, but these demonstrated limited success.

At referral Helena had been prescribed stimulant medications for ADHD and melatonin for sleep onset problems. Initial referral to CAMHS was to consider psychotropic medication for her distress and challenging behaviour. Unlicensed (off-label) use of psychotropic medication at that young age was not supported by the CAMHS psychiatrist. However, Helena's adoptive mother took her to a private paediatrician, who prescribed various medications, including propranolol, sertraline and aripiprazole.

Helena's difficulties were understood as related to co-existing conditions of ASD and developmental trauma. Although these were not mutually exclusive, the aspects of developmental trauma were predominant as relational insecurity was contributing to her ‘fight or flight’ responses to situations. There was no convincing benefit from the prescribed medications.

At the time of the report, Helena's adoptive mother was engaging with therapeutic support from the clinical psychologist in the CAMHS LAC team. This intervention was trauma-informed and systemic and it appeared to have helped the mother to shift the focus from ‘fixing’ her child to a position of acceptance and relational safety.

### Emily

Emily is a 12-year-old girl who was first referred to CAMHS in February 2019. She was the youngest of five children, all of whom were either in care or care leavers/adults.

In her birth family, before coming into care, she was the main carer for her niece and nephew while living with her adult sister from a young age, because her mother was severely ill. Emily had lived with her grandparents and then her older sister before entering foster care in 2017.

Emily described being made to feel invisible by her birth mother, resulting in her cutting off contact. She talked about feeling responsible for her past experiences and those of her family. She revealed that her brother had used her as a ‘punching bag’ and she had witnessed him trying to strangle her sister on the bed with a tie. This resulted in Emily calling the police, which ultimately led to the removal of her niece and nephew. She discussed feeling like a failure for being unable to make her family better.

At the time of her presentation, she had started self-harming by scratching her arms. She would leave notes for her foster carers relaying messages about how she was struggling and did not want to ‘be around’ anymore. The recent loss of contact with her niece and nephew, who had been taken into care without a goodbye, had apparently precipitated the start of the self-harming behaviour.

By May 2019 the notes and self-harm had reduced, but Emily had become aggressive in tone. She strove for independence and was known often to push away her carers or friends.

In a review in November 2019 Emily stated that the loss of the nephew and niece into care had felt like a loss of her own children, a feeling potentially linked to her role caring for them. When she went into care herself it was felt by CAMHS that she struggled with the feeling of losing control, possibly triggering some of her self-harming behaviour. Owing to Emily's difficulties with emotion regulation her foster carers could not engage with her anymore. She nevertheless continued to use the notes left outside her bedroom to communicate her feelings. She would often not want to talk about her birth family, saying she did not want to get angry by remembering them.

Emily on one hand believed herself to be mature enough to look after others, but on the other hand had difficulties with her own hygiene, had toddler-like outbursts and continued to use the notes to communicate, rather than expressing her distress verbally. Taking into consideration her difficulties, a referral to dramatherapy was made.

In August 2020 her foster family placement was made permanent. In November a new child joined the foster family, which resulted in Emily self-harming again for the first time in 18 months. Emily seemed to be angry about sharing her primary caregiver with anyone else.

Emily cared for her dolls as though they were real. It seemed that she wanted to escape into this idealised world, a way to undo the past events she felt responsible for. She would try to parent the new foster child. She would try to manage other children in class at school.

At the time of writing, DDP-informed therapy sessions were suggested, and a request had been made to the social worker to find out more about Emily's early story to assist with therapeutic life story work.

## Discussion

These real-life cases present examples of trauma-informed approaches and inter-agency intervention. To reflect on these points, we have grouped them into the following themes of when things have gone well in these domains and when they have not.

### When things go well: the benefits of a trauma-informed approach

Exposure to trauma is a common experience among looked after children. These three case studies all demonstrate specific examples of how maladaptive behaviours can stem from early traumatic experiences of abuse, neglect or loss.

A systematic review by Denton et al raised the problem of current diagnostic methods.^9^ It highlighted several papers that discussed how children with developmental trauma are often given multiple discrete comorbid diagnoses resulting in several treatment plans that do not tackle the developmental issues that gave rise to these, often interrelated, clusters of symptoms.

In all three cases there are examples of emotional distress leading to threatened or real high-risk behaviour such as self-harm, suicide attempts and aggression. In all three cases this was identified as a manifestation of emotion dysregulation, which can be a long-term effect of traumatic experiences during the early years as the young person does not learn more adaptive approaches to handling emotions. They are left, as van der Kolk refers to it, as having ‘deficits in emotional self-regulation’.^10^

In Sarah's case, it was also understood to be a way of verifying her attachment by evoking strong emotions from her caregiver and triggering an urgent response and reassurance each time an attempt at suicide was made. This was further demonstrated in the way she consistently made her caregiver aware of what she had done.

In Emily's case we saw her need to mother her niece and nephew as her way of regaining control in a perceivably uncertain world while on the other hand manifesting her own emotional need through self-harm and notes outside her bedroom to seek response and care. This manifestation was Emily's attempt to gain control in order to feel safe in her uncertain and unsafe world.

In Helena's case, multiple comorbid diagnoses were assigned, leading to several pharmacological and non-pharmacological therapies at various times that had limited success. Helena's case presents an example of where potentially a trauma-informed formulation would have allowed her difficulties to be looked at as a collection of interlinked symptoms resulting from early exposure to chronic severe neglect and witnessing or enduring adverse and traumatic events, instead of multiple comorbid diagnoses.

In each of these cases we see a pattern of emotion dysregulation leading to a spectrum of symptoms, all of which can be linked in some way to early trauma and insecure attachment.

As is often the case in healthcare and especially in psychiatry, the diagnostic category given to an individual is like a key: it unlocks the door to some treatment pathways but not others, depending on the category assigned. Each of these case studies has examples of other diagnoses being used in place of or preceding a developmental trauma approach.

Considering these patterns of symptomatology, each of these case studies demonstrates how sometimes manifestations of the impact of early trauma can be ‘misdiagnosed’ as multiple comorbid conditions that confer treatments that inevitably fail to resolve the crucial underlying issues that could be identified through formulation exercises as narrated above. Developmental trauma as a therapeutic approach therefore allows us to consider the spectrum of presentations that trauma can lead to, respond to them and treat appropriately.

In each of the cases above, an attachment- or trauma-focused intervention such as DDP was used years after presentation of the initial difficulties that brought the child and family to the attention of CAMHS. Therefore, the advantage of recognising a formulation or diagnosis of developmental trauma is that young people would get correct and early intervention, moving beyond the classic linear paradigms of traditional CAMHS treatment.

### When things go well: reflections on multi-agency working

In Sarah's case, inter-agency working was crucial to decision-making when in-patient care was considered. The joined-up work between CAMHS and social services allowed a shared perspective on the utility of previously tried treatments (CBT, DBT and an antidepressant) and collaborative consideration of underlying issues gave a holistic picture and joint assessment of risks that helped avoid an unnecessary and potentially detrimental in-patient admission.

The importance of this multi-agency approach has been highlighted in a recent paper reporting that when looked after children were treated with a combined direct therapy and indirect multi-agency consultation approach compared with purely one or the other, a significantly greater improvement in SDQ scores was seen.^11^ This was in spite of those who received both therapies presenting with more severe presentations.

### When things go wrong: reflections on multi-agency working

In all these cases we see examples of inter-agency working: its positive aspects and its challenges when CAMHS are at the centre of a ‘hub and spoke’ model in which they advise carers and professionals on key aspects of the child's functioning and on therapeutic approaches to respond to behaviours that are often the result of trauma.

In Helena's case, the distribution of work between multiple agencies and the additional spread of interventions between public and private providers led to a vast therapeutic landscape that was not fully cohesive. It seems that one team focused on psychological therapies whereas the other took a more pharmacological approach.

In Emily's case there were challenges for interagency working where parts of the team felt under pressure. For example, there was a delay to the therapeutic life story work that the CAMHS team deemed important for her recovery, due to the perceived delay in getting the necessary background information for this from the social workers. This can potentially lead to splitting within the system and can be a barrier to delivering successful multi-agency intervention for children with complex mental health presentations.

### Implications

We propose that the use of a developmental trauma approach and multi-agency working can improve clinical outcomes for young people with adverse childhood experiences who present with mental health difficulties. We also offer learning points for psychiatrists and other health and care professionals working with individuals with a history of early adverse experiences. Particularly when considering those with a history of significant early trauma, we hope this paper has demonstrated the utility of a developmental trauma approach and the benefits of multi-agency working to move beyond a paradigm in current CAMHS of diagnostic boxing and linear treatment pathways in which trauma is often seen as irrelevant.

## Data Availability

The data that support the findings of this study are available on request from the corresponding author, N.M. The data are not publicly available due to their containing information that could compromise the privacy of research participants.

## About the authors

**Nic Miller** is a medical student in the School of Medicine at the University of Nottingham, UK. **Sreekumar Nair** is a consultant psychiatrist with Nottinghamshire Healthcare NHS Foundation Trust, Nottingham, UK. **Pallab Majumder** is an honorary associate professor at the University of Nottingham and a consultant psychiatrist with Nottinghamshire Healthcare NHS Foundation Trust, Nottingham, UK.
